# Mouse auditory cortex sub-fields receive neuronal projections from MGB subdivisions independently

**DOI:** 10.1038/s41598-024-57815-3

**Published:** 2024-03-25

**Authors:** Chi Wang, Zhen-yu Jiang, Jian-yuan Chai, Hong-suo Chen, Li-xia Liu, Tong Dang, Xian-mei Meng

**Affiliations:** 1 Inner Mongolia Institute of Digestive Diseases, The Second Affiliated Hospital of Baotou Medical College, Baotou, China; 2 Department of Gastroenterology and Hepatology, The Second Affiliated Hospital of Baotou Medical College, Baotou, China; 3 Department of Scientific Research, The Second Affiliated Hospital of Baotou Medical College, Baotou, China

**Keywords:** Mouse auditory cortex, Neuronal circuits, Belt area, Thalamus projection, Optical imaging, Thalamus, Cortex, Auditory system, Neuroimmunology, Neuronal physiology

## Abstract

Mouse auditory cortex is composed of six sub-fields: primary auditory field (AI), secondary auditory field (AII), anterior auditory field (AAF), insular auditory field (IAF), ultrasonic field (UF) and dorsoposterior field (DP). Previous studies have examined thalamo-cortical connections in the mice auditory system and learned that AI, AAF, and IAF receive inputs from the ventral division of the medial geniculate body (MGB). However, the functional and thalamo-cortical connections between nonprimary auditory cortex (AII, UF, and DP) is unclear. In this study, we examined the locations of neurons projecting to these three cortical sub-fields in the MGB, and addressed the question whether these cortical sub-fields receive inputs from different subsets of MGB neurons or common. To examine the distributions of projecting neurons in the MGB, retrograde tracers were injected into the AII, UF, DP, after identifying these areas by the method of Optical Imaging. Our results indicated that neuron cells which in ventral part of dorsal MGB (MGd) and that of ventral MGB (MGv) projecting to UF and AII with less overlap. And DP only received neuron projecting from MGd. Interestingly, these three cortical areas received input from distinct part of MGd and MGv in an independent manner. Based on our foundings these three auditory cortical sub-fields in mice may independently process auditory information.

## Introduction

Hearing plays an important role for the survival of animals. Animals recognize intensities, directions, frequencies, and even emotional significance of sound^1,2^. The auditory cortex plays an essential role in hearing. Previous studies have shown there are six sub-fields in mouse auditory cortex: primary auditory field (AI), secondary auditory field (AII), anterior auditory field (AAF), ultrasonic field (UF), dorsoposterior field (DP), and insular auditory field (IAF)^3–6^. Although all these cortical sub-fields respond to acoustic stimuli, it is unclear currently whether these areas work cooperatively or independently. As the first step to explore the role of auditory cortical sub-fields, here we focused on their thalamo-cotical connections. The auditory cortex mainly receives inputs from the medial geniculate body (MGB) of the thalamus. MGB is histologically divided into three principal subdivisions: ventral nucleus (MGv), dorsal nucleus (MGd), and medial nucleus (MGm) numerous species, including humans^7–9^. Information on whether auditory cortical sub-fields receive input from the same or different parts of MGB may help us to understand how auditory cortical sub-fields contribute to hearing.

In addition, previous studies demonstrated that AII received projections from MGv and DP receives projections from MGd in mice by flavoprotein fluorescence Imaging identification and tracer injection^10,11^. Compared with optical imaging data, we found the some positions of flavoprotein fluorescence identified auditory cortex sub-fields were different. To examine the difference between our research and previous studies, belt areas (UF, AII and DP) were focused.

There are two thalamo-cortical (TC) pathways: lemniscal and nonlemniscal^12,13^. The lemniscal TC pathway mainly arises from the MGv and projects to the AI and other tonotopic auditory cortical areas^2,14–16^, while the nonleminiscal TC pathway mainly arises from the MGm and MGd and projects to the nonprimary auditory cortex^12,17,18^. Recent studies have demonstrated that AAF and AI, and IAF in mice receive inputs from MGv in an independent manner^6,19^. However, the thalamic origin of auditory input to UF, AII and DP has not been fully clarified. To address which subdivision of the MGB projects to these areas (UF, AII and DP), and to test whether these areas receive common input or not, we investigated auditory TC connections retrograde tracer injection in this study.

Mouse brain atlas and stereotaxic injection were very popular in most auditory research, by using optical imaging that a physiological areal identification method may support precise areal location on cortex surface.

## Materials and methods

### Animals

Fifty-six of 6 weeks old male C57BL/6J mice (Saiye Biotechnology Co., Suzhou, China), were used for the experiments. All animals were kept in an animal room with 12 h light/dark cycle and food/water available ad libitum. All experiments were approved by the Committee for Animal Experiments of Baotou medical college, and performed in accordance with the Guidelines for Use of Animals in Experiments of Baotou medical college. Experimental procedures followed previous study^4,6^.

### Animal preparation

Briefly,** t**he mixture of ketamine and xylazine (initial dose: ketamine 80 mg/kg, xylazine 8 mg/kg; supplemental doses: ketamine 40 mg/kg/h, xylazine 4 mg/kg/h) were used to anesthetized animals. Dexamethasone (2.5 mg/kg) were injected after anesthesia to reduce cerebral edema. Atropin (atropium sulfuricum, 0.25 mg/kg, subcutaneously) was injected to reduce mucous secretion in the respiratory tract. All drug were supported by Baotou medical college. Anesthetized mouse was placed in a stereotaxic holder with heating pad maintaining rectal temperature around 36 °C. A metal screw was pasted to the skull to hold the head of animal during recording experiment. To ensure the whole auditory cortex was exposed, the skull over the left auditory cortex where in the rostro-caudal direction approximately 5 mm and dorso-ventral direction approximately 4 mm was removed and the exposed dura mater was also resected completely. The dorsal edge of the exposed cortex was made parallel to the midline, and was used to indicate the rostro-caudal direction of the recording auditory field.

### Voltage sensitive dye optical imaging

**Figure 1:**
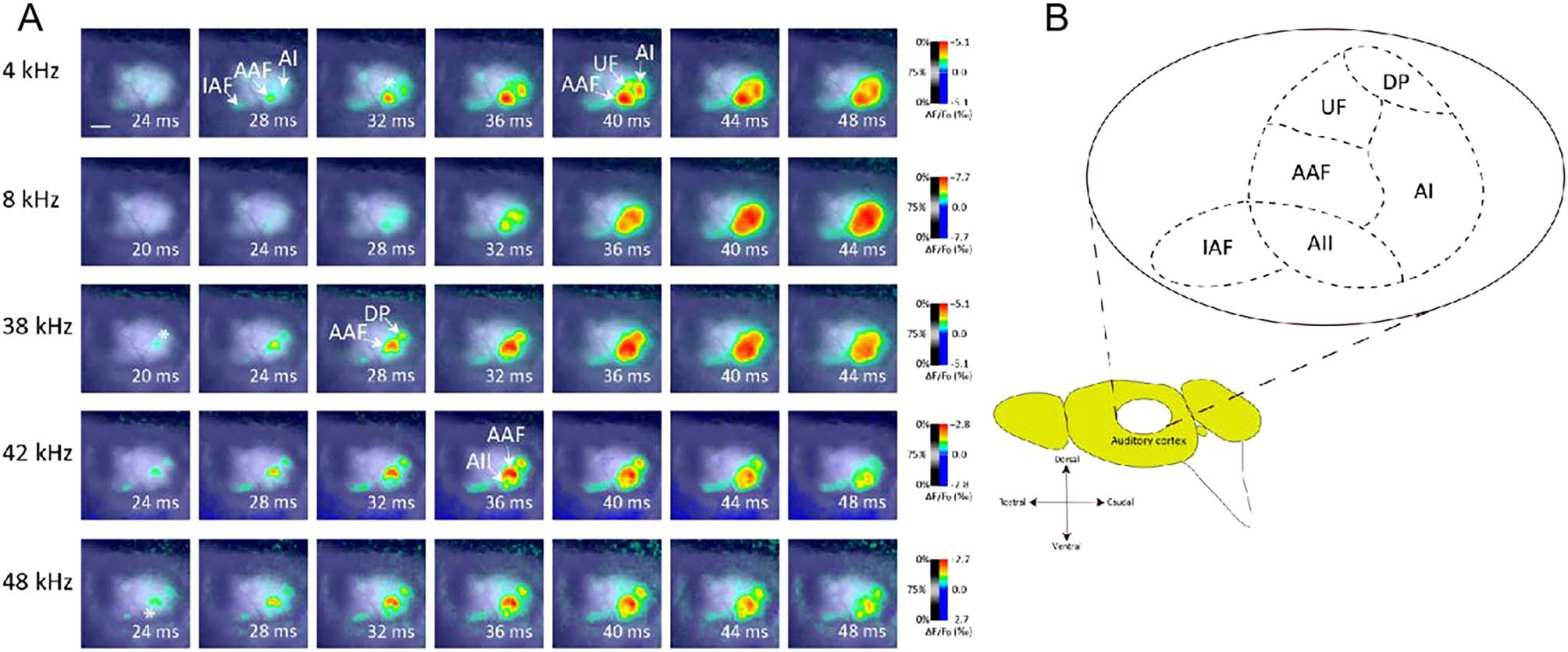
Identification of belt areas in mouse by optical imaging in vivo. (**A**) Time course of auditory response in cortex. Responses to different frequency tone at each time are shown by colors according to the color bar on the right. Belt areas were responded by different sound stimulation. Scale bar was 1 mm. (**B**) Relative position of auditory field in mice. According to imaging results and previous reports, we drew this schematic figure of auditory cortical sub-fields. From this figure the position of UF, AII, and DP can be confirmed.

The exposed cortex was stained with the voltage-sensitive dye RH-1691 (0.6 mg/ml in saline; Optical Imaging Ltd, Rehovot, Israel) for 60 min. After staining, the cortex was washed and covered with saline (0.7 mg/ml). Because of the small size of mice auditory sub-fields and the lack of histological features of each sub-field, Optical imaging was carried out to determine the position of auditory sub-fields (Fig. 1). Optical signal were detected with high resolution complementary metal oxide semiconductor imaging system (100 × 100 pixels; MiCAM Ultima, BrainVision, Tokyo, Japan) was used to catch optical signals at a sampling interval of 1.0 ms. The principles of the imaging system have been described previously^20,21^. The sensor array covered an area of 6.25 × 6.25 mm^2^ in this optical system. The area covered by one pixel was 62.5 × 62.5 μm^2^ approximately. As acoustic stimuli, pure tones (4, 8, or 16 kHz, 50 ms duration, 10 ms onset and offset cosine ramps, 60 dB sound pressure level) were produced and delivered with TDT system 3 hardware and software (Tucker-Davis Technol- ogies, FL, USA), and were applied to the right ear of the animal.

Fractional fluorescence signals (DF/F0) were calculated and averaged for 20 consecutive recordings for each tone frequency, then encoded in colour and superimposed on the cortical surface with custom-made software^20^. Onset response to a tone in the A1 and AAF was defined as the binarised response using a threshold of 30% of the maximum response (Fig. 1D). A1 and AAF were identified using the frequency gradient determined by the onset responses to 4, 8, and 16 kHz tones, as in previous studies^4,6^.

Using Epi-illumination from a tungsten–halogen lamp to evoke the voltage-sensitive dye (excitation filter: λ = 632 ± 11 nm; dichroic mirror: λ = 650 nm), and the fluorescence through an absorption filter (λ > 665 nm) was recorded with a CMOS imaging system (MiCAM Ultima, Brainvision, Tokyo, Japan), at a sampling interval of 1 ms. Recordings of 256 frames were obtained; fractional fluorescence signals (ΔF/F0) were counted using the average of 50 frames before the stimulus onset as F0. Twenty continuous recordings were then averaged for each tone frequency, and the average signal was encoded in color and superimposed on the cortical surface image by using custom-made software (MiCAM Analyzer)^20^. Onset response (OR) to a tone in a cortical field was determined by binarizing all responses with a threshold of 20% of the maximum response, again using that custom-made software, and was used as a position guide for tracer injection into the target area.

### Tracer injections

The core auditory cortical sub-fields (IAF, AAF, and AI) of mouse showing response firstly after acoustic stimulation. These areas can be identified by their characteristic frequency gradient (Fig. 1A). Except these three areas, in dorsal and ventral part of AAF two areas were identified independently, UF and AII. And there also has a region can responded by sound stimulation in dorsal part of AI which is DP. After cortical area identification, tracer injection experiments were carried out by using Alexa Fluor 555- and Alexa Fluor 488-conjugated cholera toxin subunit B (red-CTB and green-CTB, respectively, 0.5% in 0.1 M phosphate buffer [PB]). Double injection experiments were performed that red-CTB was made into the center of the AII, green-CTB was made into the sites of UF respectively. We chose the position of onset response (OR) on the brain surface image to confirm the position of injection. The injections were conducted by air pressure. For pressure injections, glass micro-pipettes (tip diameter 30 μm) were used to inject 0.1 μl of tracer solution over 5 min approximately. During the injections, glass micro-pipettes were penetrated into the cortex surface to layer IV (about 300 μm) a certain depth receiving thalamus inputs. After injection, mouse was sutured the wound and continuously fed in home-cage.

### Histochemistry

After a 3 days survival period, animals were perfused with 4% paraformaldehyde in 0.1 M PB (pH 7.4) during deep anesthesia (ketamine 120 mg/kg and xylazine 12 mg/kg). The brains were cryoprotected with 15% and 30% sucrose, and cut into 40 μm-thick serial coronal sections by a freezing microtome. Brain sections were subjected to immunohistochemistry with SMI-32 (Neurofilament H monoclonal antibody) to identify the subdivisions of MGB. For the immunohistochemistry, a SMI-32P (1:2000, diluted by goat serum blocking buffer) was used as the primary antibody, and Biltinylated goat anti-mouse IgG (1:200, diluted by blocking buffer) was used as the secondary antibody. After blocking for 1 h at room temperature (RT) in phosphate-buffered saline containing 5% goat serum and 0.3% Triton X-100, sections were incubated with a primary antibody in the blocking solution overnight at 4 ℃. The sections were then incubated with a secondary antibody in the blocking solution for 1.5–2 h at RT. Then Diaminobenzidine (DAB) solution was used for up to desired intensity. Stop the reaction with 4% PFA in 0.5 M PB for 10 min and then rinse sections with PBS. Finally we mount the sections on glass slides and cover-slip them with Mowilo 488. For other experiments, all sections were stained with DAPI (4,6 diamidino 2 phenylindole) for distinguishing layers in cortex.

### Data analysis

Labeled cells were visualized with a fluorescent microscope (IX71, Olympus, Tokyo, Japan) and recorded with a digital camera (DS-Fi2, Nikon, Tokyo, Japan). Photo images of different labeled cells were merged by using Photoshop CS3 12.0.4 (Adobe Systems, San Jose, CA). The location of labeled cells was determined by referencing to the mouse brain atlas (Franklin and Paxinos, 1997). The position of labeled cells was measured with Adobe System. Data are represented as means ± standard deviation (SD).

### Ethical approval and consent to participate

This study and included experimental procedures were approved by Committee for Animal Experiments of Baotou medical college (Approval NO. LW-004). All experiments were approved by the, and performed in accordance with the Guidelines for Use of Animals in Experiments of Baotou medical college. Because this experiment did not concern clinical data there is no consent to participate.

## Results

### Identification of MGB cells projecting to belt areas

To examine the detail positions of MGB neurons which projecting to these belt areas, retrograde neuronal tracer was injected after mapping these areas in the cortex. As a result, belt areas (UF, AII and DP) had no tonotopy (Fig. 1A), which means the position of belt areas unlike core areas (IAF, AAF and AI) respond to changing OR position with the tone frequency gradient. By using this method, the positions of belt areas (UF, AII and DP) were marked by recording OR to pure tones at 4-, 32-, and 38-kHz. According to the ventro-rostral to dorso-caudal tonotopic gradient AAF and its caudal core area AI was identified. Previous studies showed similar tonotopy of AAF and AI^22,23^. According to a previous studies^5,23^ UF was located in the dorsal of AAF, region in the ventral of AAF was AII, and DP located in the dorsal of AI, three belt areas were identified (Fig. 1A,B).

**Figure 2:**
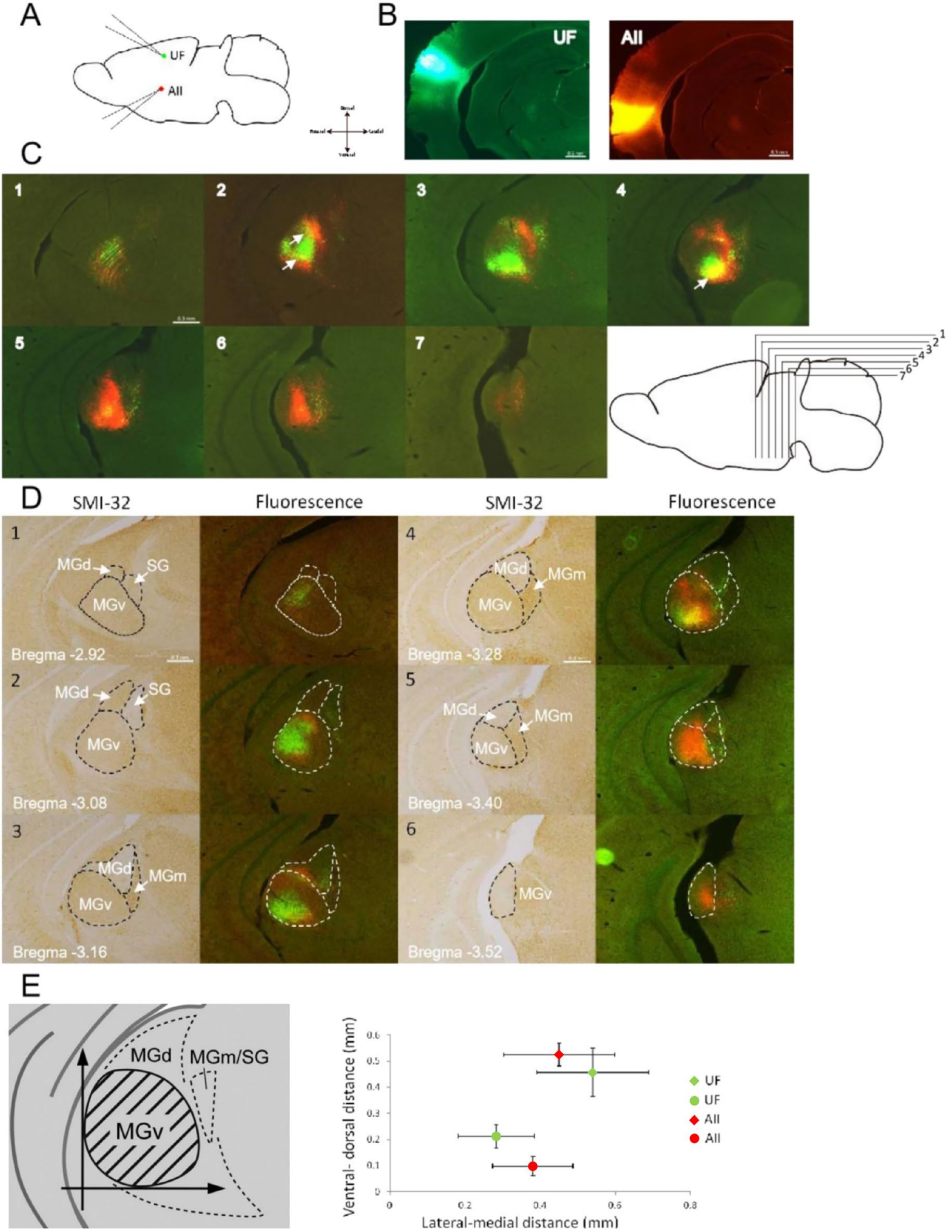
Retrogradely labeled cells were found in MGB. (**A**) Schematic drawing of tracer injection. According to Optical imaging result, belt areas (UF, AII, and DP) can be identified and 0.1 μL green-CTB and red-CTB were injected into UF and AII separately. (**B**) Photos of coronal sections with the tracer injection site stained with DAPI under florescence microscope. Scale bar was 0.5 mm. (**C**) Photos of a DAPI-stained coronal section including the MGB which be merged fluorescence images of retrograde labeling (red and green). The interval distance between two sections is 120 μm as right drawing. (**D**) Photos of coronal sections stained with SMI-32. Dot line indicates the boundary of each MGB subdivision. (**E**) Quantitative analyses of the position of retrogradely labeled cells in the MGB. Left: Schematic diagram illustrating the coordinate for quantifying the position of retrogradely labeled cells. A ventrodorsal line passing through the most lateral edge of MGv served as the vertical axis, and a rostrocaudal line passing through the most ventral edge of MGv served as the horizontal axis. Right: Averaged position of labeled region in MGB. Red-points and green-points indicate the center of labeled region by double injection in UF (green) and AII (red) which were calculated by 16 individual animals. Squares reflect labeled cells in MGv and circles reflect labeled cells in MGd; error bars were SD.

The photos of sections with injection site were shown (Fig. 2A,B). Comparing with scale bar, the diameter of injection sites were less than 0.5 mm (Fig. 2B) indicating that the tracers were confined to the target areas without diffusion. Retrogradely labeled cells were identified in MGB by CTB, indicating that tracers works well (Fig. 2C). Except labeled cells, some vague labeling without obvious cellular profiles were also found in the MGB, indicating there was some anterograde labeling of corticothalamic axon terminals by CTB. To reveal the projection from MGB neuron to cortex, we only focused on retrogradely labeled cell bodies in the following analysis. In these sections containing the MGB, red labeled cells (UF-injection), green labeled cells (AII-injection), and the yellow region in both (UF and AII) were observed, indicating neurons in MGB project to UF and AII; portion of UF and AII received common input from MGB. To examine the accurate position of labeled cells in MGB subdivision, immuno-stained the sections (same animal) that contained labeled cells with an SMI-32 antibody were presented for comparison (Fig. 2D). As reported previously^24,25^, MGv was more strongly stained with SMI-32 than MGd. MGB subdivisions revealed by SMI-32 staining largely agreed with mouse brain atlas. Immuno-stained result clearly showed the location of the cluster of retrogradely labeled cells corresponded to a ventral part of the MGv and that of MGd (Fig. 2D). This results indicated that UF and AII received projection from a ventral part of MGv and that of MGd. MGv-AII projection was agreeble to the previous study^11^. In order to quantitative analyses of retrogradely labeled cells in MGB, a ventraodorsal line as the vertical axis and a rostrocaudal line as the horizontal axis were drawn (Fig. 2E left). The center of labeled regions were chosen as red points and green points. The distance between points to axis reflects the center of labeled region to the vertical or horizontal axis. Similar observations were obtained in all 16 animals that we examined (Fig. 2E). Along the borders between red-CTB labeled and green-CTB labeled regions in the ventral part of MGv and that of MGd, a few double labeled cells were also shown (Fig. 2B, arrowhead), regardless of non-overlapped tracer injections between UF and AII. This result suggests that these double labeled cells (yellow region) may project to both UF and AII.

**Figure 3:**
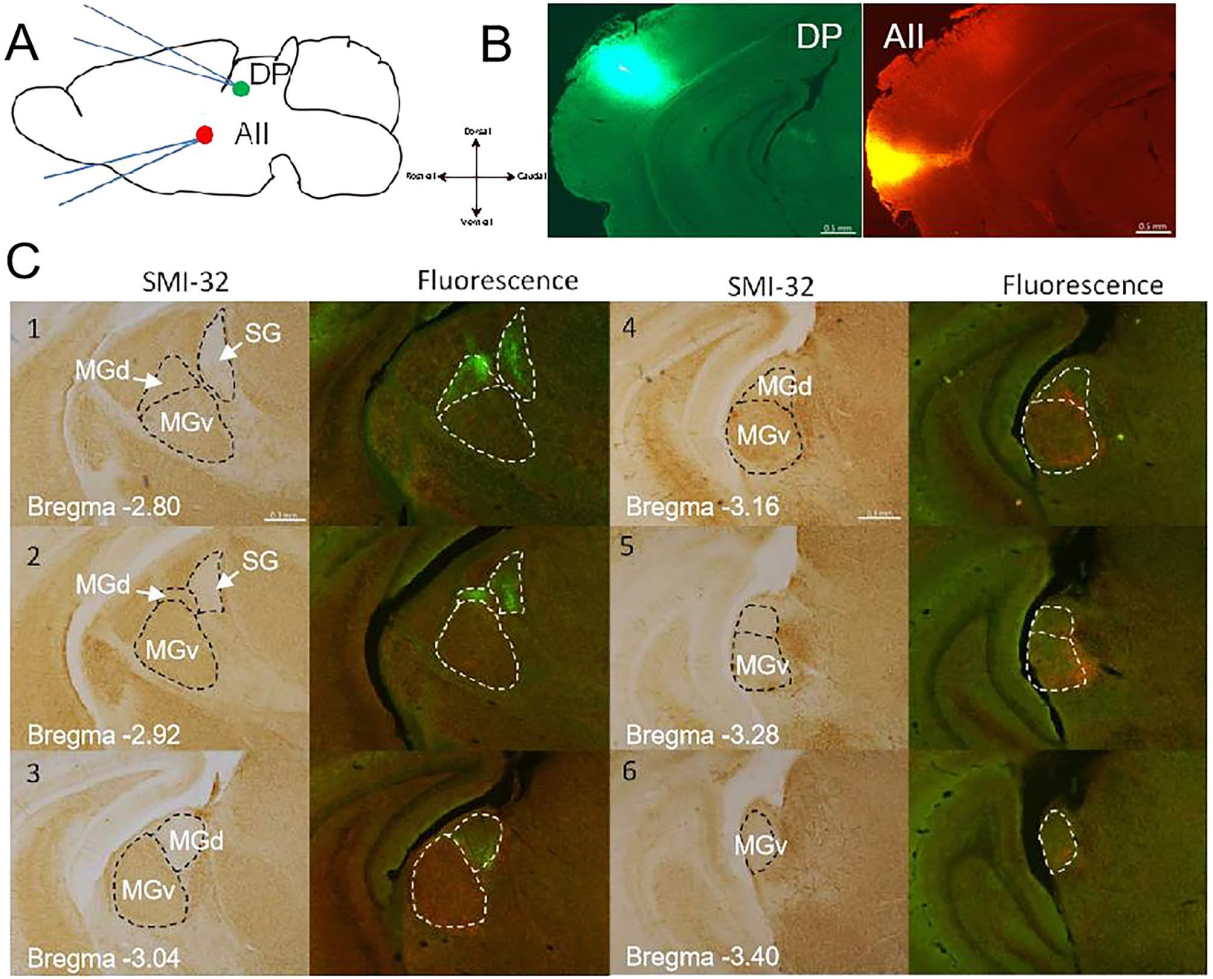
Retrogradely cells were found in MGB. (**A**) Schematic drawing of double tracer injected into the DP and AII. (**B**) Injection site of DP and AII. (**C**) Photos of coronal sections which is stained with SMI-32. Dot line indicates the boundary of every MGB subdivision. DP-projection cells were located in MGd and SG.

Double injection experiment was also presented to DP and AII, respectively (Fig. 3A,B), DP-projecting green-CTB-labeled cells were found in MGd and Suprageniculate nucleus (SG) (Fig. 3C). Unlike double-labeling for UF and AII, there were fewer double labeled cells in the ventral part of MGd. This result suggests that neurons in MGd and SG project to DP. The projection result of DP was similar to the previous study^10^.

### Existence of MGv cells projecting to high-order auditory sub-fields

**Figure 4:**
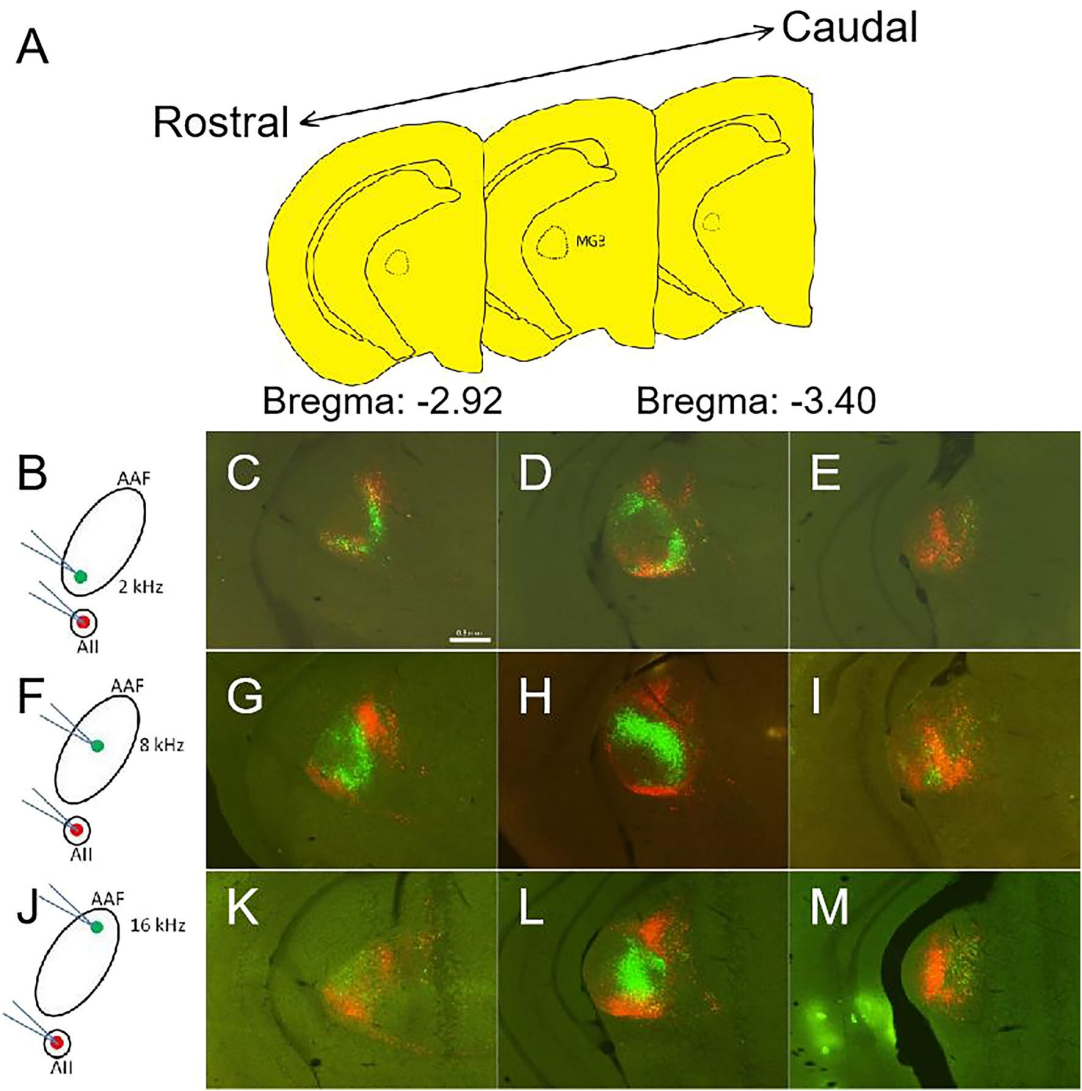
Distributions of labeled cells in MGB along the rostrocaudal axis. (**A**) Schematic drawing of three coronal sections which queued from rostral to caudal part of MGB, respectively. The interval distance between two sections is 240 μm. (**B,F,J**) Schematic drawing of double injection in AAF and AII separately. (**C–E**) Rostrocaudal distribution of retrogradely labeled MGB cells after double tracer injections into two sites with OR to 4-kHz (green) in AAF and 34-kHz (red) in AII. (**G–I**) Retrogradely labeled MGB cells from rostral to caudal after double tracer injections into two sites with OR to 8-kHz (green) in AAF and 34-kHz (red) in AII. (**K–M**) Rostrocaudal distribution of retrogradely labeled cells in MGB after double tracer injections into two sites with OR to 16-kHz (green) in AAF and 34-kHz (red) in AII. Scale bar in (**C**) is 0.3 mm.

Previous studies reported that the MGv projected to the AI and AAF^14,26^, and IAF^6^. Our results showed that the MGB neurons that project to the UF and AII were both located in MGv and MGd (Fig. 2C), although DP received thalamic input from MGd and SG (Fig. 3C), raising a possibility of the existence of the MGv neurons that commonly project to high frequency responsed auditory cortical sub-fields except for DP. In addition, it was still unclear whether the neurons in MGv that projected to core areas and those in belt areas were the same part or not. AAF is the largest auditory cortical sub-field in mice and the position is close to UF and AII, so we focused on it firstly. To answer this question, red-CTB and green-CTB were injected into the AII and AAF respectively. Because the length of AAF was about 1 mm, the diameter of injection site was less than 0.5 mm. The tracer could not cover the whole AAF by single injection, so this experiment was completed in three phases (Fig. 4B,F,J). The injection sites of AAF were selected at the position of OR to 4 kHz, 8 kHz and 16 kHz which corresponded the lower, middle, and upper part. The injection site of AII was chosen at the site of OR to 32 kHz. In coronal sections containing MGB (Fig. 4A), we found two kinds of fluorescent signals (Fig. 4B–M). When green-CTB was injected into the 4-kHz site that corresponds to the ventral part of AAF. Green-CTB labeled cells were located in a ventromedial part of the MGv and red labeled cells located in a ventral and dorsal part of the MGv (Fig. 4C–E). Although there were several double labeled cells, a large number of labeled cells were found in different portions in MGv (Fig. 4C–E). The double labeled cells might be caused by tracer diffusion in injection site (the distance between two injection sites was about 0.4 mm less than that of injection diameter). Use the same method green-CTB was injected into the middle part (8-kHz) or dorsal part (16-kHz) of AAF in other animals (Fig. 4F,J). Almost no double labeling was seen (Fig. 4G–I,K–M). Major clusters of red-CTB–labeled cells were found in the ventral and dorsal part of MGv (Fig. 4L) and it was consistent with our previous result (Fig. 2C arrowhead). Most labeled cells distributed in MGB in all experiments were single labeled, but only green-CTB were injected into upper part of AAF where in higher frequency trail have a small cluster double labeled cells in MGv (Fig. 4L). These results suggest that AAF where response in higher frequency part may receive common input with AII. Although when double tracers were injected into the lower part of AAF (where response in low frequency) and AII, double labeled cells were found in ventral part of MGv. It might be caused by tracer diffusion in injection sites.

**Figure 5:**
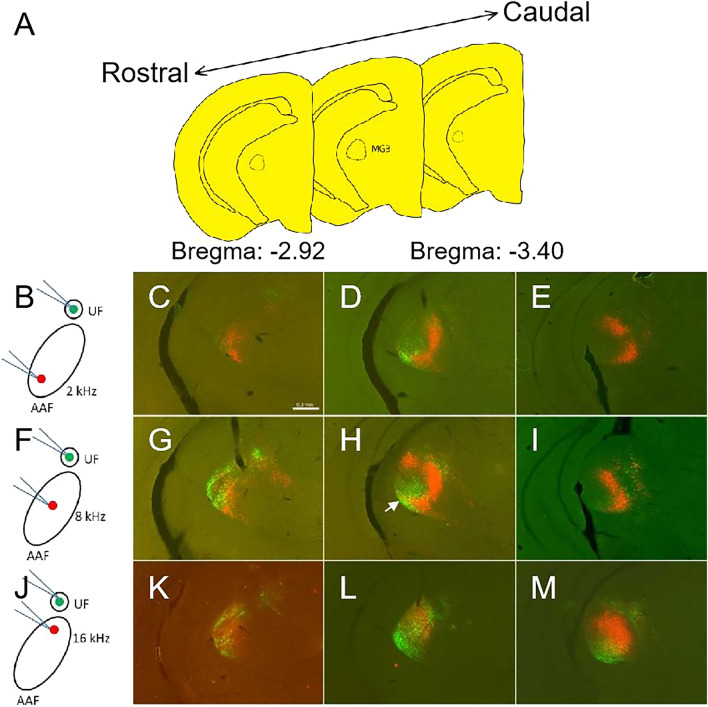
Distributions of labeled cells in MGB along the rostrocaudal axis. (**A**) Schematic drawing of three coronal sections from rostral to caudal part of MGB, respectively. The interval distance between two sections is 240 μm. (**B,F,J**) Schematic drawing of double injection in AAF and UF separately. (**C–E**) Rostrocaudal distribution of retrogradely labeled MGB cells after double tracer injections into two sites with OR to 4-kHz (red) in AAF and 4-kHz (green) in UF. (**G–I**) Retrogradely labeled MGB cells from rostral to caudal after double tracer injections into two sites with OR to 8-kHz (red) in AAF and 4-kHz (green) in UF. (**K–M**) Rostrocaudal distribution of retrogradely labeled cells in MGB after double tracer injections into two sites with OR to 16-kHz (red) in AAF and 4-kHz (green) in UF. Scale bar 0.3 mm in (**C**).

**Figure 6:**
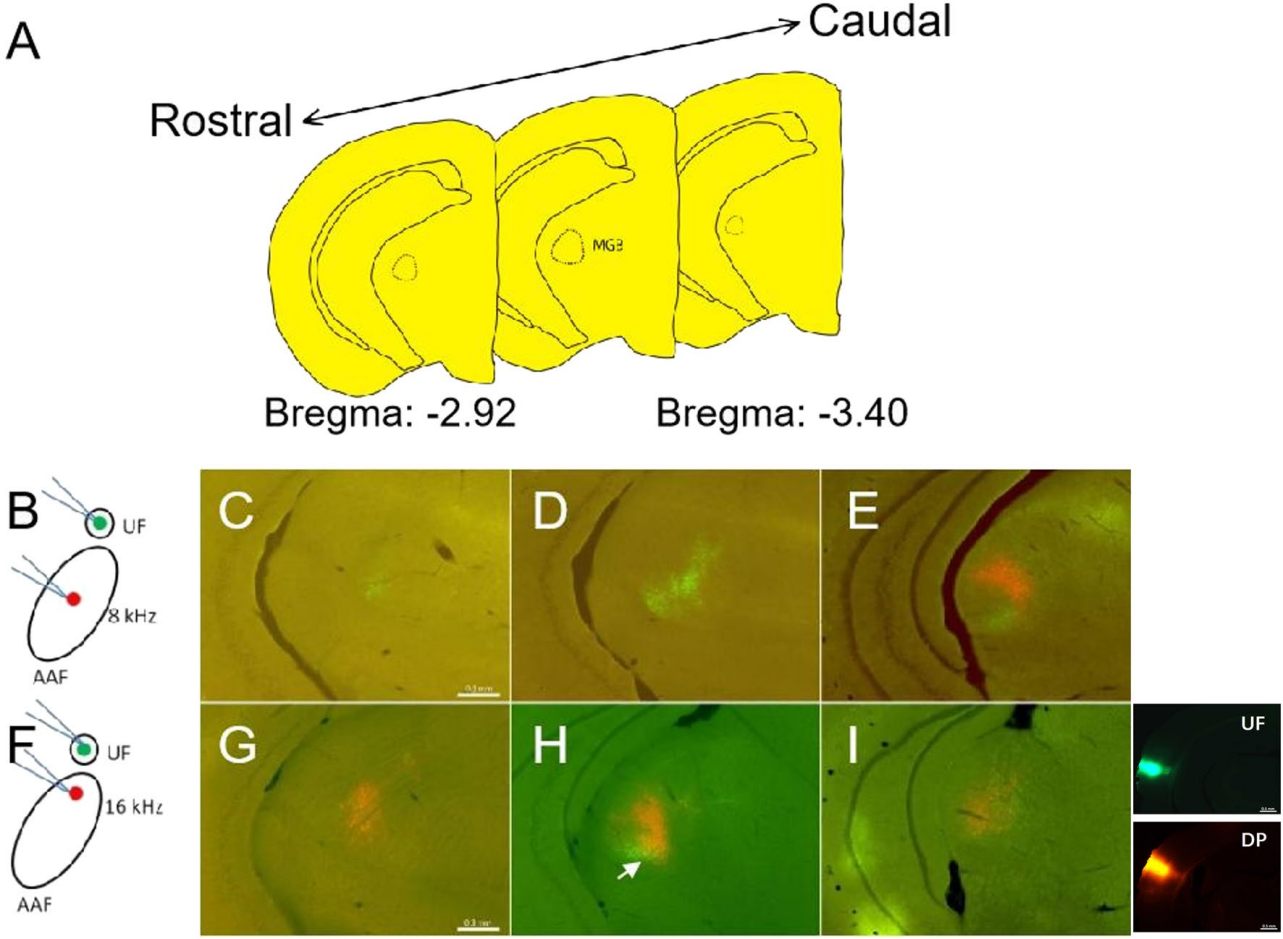
Distributions of labeled cells in MGB along the rostrocaudal axis. (**A**) Schematic drawing of three coronal sections from rostral to caudal part of MGB, respectively. The interval distance between two sections is 240 μm. (**B,F**) Schematic drawing of double injection in AAF and UF separately. (**C–E**) Rostrocaudal distribution of retrogradely labeled MGB cells after double tracer injections into two sites with OR to 8-kHz (red) in AAF and 4-kHz (green) in UF. (**G–I**) Retrogradely labeled MGB cells from rostral to caudal after double tracer injections into two sites with OR to 16-kHz (red) in AAF and 4-kHz (green) in UF. Photos on the lift side of I were injection sites of UF and DP. Scale bar 0.3 mm in (**C**) and scale bar 0.5 mm in injection site photos.

Based on the experiments above, AII and AAF high frequency response onset receiving neuronal input from the ventral part of MGv, it suggested that this part of MGv may project to high-order area of mouse auditory cortex. Because of UF also receiving neuronal input from the ventral part of MGv, we choose the closer core area AAF to investigate whether there is common input or not. As shown in Fig. 5B,F,J the injection sites of AAF were also selected at the site of OR to 4 kHz, 8 kHz and 16 kHz. The injection site of UF was chosen at the site of OR to 4-kHz. In the coronal sections containing the MGB (Fig. 5A), there were almost no double-labeled cells in UF and AAF-2 kHz injection experiment (Fig. 5B–E). However, when green-CTB was injected into UF and red-CTB was injected into 8 kHz or 16 kHz-AAF (Fig. 5F–M), some retrogradely double-labeled cells were found in the ventral part of MGv (Fig. 5H arrowhead, M). To quantify the distribution of labeled cells in MGB, the number of labeled cells in each subdivision was counted and shown in Table 1. The quantification analyses demonstrated the common projecting neurons mainly focus on MGd and MGv. The distances between tracer injection sites in UF and AAF, for OR sites to 8-kHz, and 16-kHz tones, were less than 0.4 mm, which might be caused by tracer spread. So control experiments which decreased the tracer volume to half were presented (Fig. 6A–I). The results showed that the number of double labeled cells was decreased in the trial of UF and AAF-16 kHz injection (Fig. 6F, Table 2) compared with the experiment above (Fig. 5J, Table 1). But still there were a few double- labeled cells. Although we controlled injection volume of CTB, there is no guarantee that this double-labeling indicates common input because of these injection sites were too close to each other. Whether UF is a part of tonotopic AAF is still arguing^23,27^, our results suggest that UF and high-order response of AAF receive less common inputs from the ventral part of MGv (Fig. 6H arrowhead). It may serve as an evidence that UF is a isolated auditory sub-field.

**Table 1:** Distribution of retrogradely labeled cells in subdivisions of the MGB (0.1 μL).

	Single labeled cells		Double labeled cells
	Injection into UF	Injection into AAF (8 kHz)	Double labeling
	N	N	N
MGd	26	117	15 (10.0%)
MGm/SG	114	10	0 (0.0%)
MGv	478	179	18 (2.7%)
Total	598	332	33 (3.5%)

	Single labeled cells		Double labeled cells
	Injection into UF	Injection into AAF (16 kHz)	Double labeling
	N	N	N
MGd	84	82	2 (1.2%)
MGm/SG	31	16	3 (6.4%)
MGv	268	106	19 (5.1%)
Total	383	204	24 (4.1%)

N is the number of labeled cells. The percent is the ratio of the number of double-labeled cells to total number of labeled cells.

*AAF* anterior auditory field, *MGd* dorsal subdivision of the MGB, *MGm/SG* medial subdivision of the MGB/suprageniculate nucleus, *MGv* ventral subdivision of the MGB.

**Table 2:** Distribution of retrogradely labeled cells in subdivisions of the MGB (0.05 μL).

	Single labeled cells		Double labeled cells
	Injection into UF	Injection into AAF (8 kHz)	Double labeling
	N	N	N
MGd	25	74	0 (0.0%)
MGm/SG	18	9	0 (0.0%)
MGv	94	97	0 (0.0%)
Total	137	180	0 (0.0%)

	Single labeled cells		Double labeled cells
	Injection into UF	Injection into AAF (16 kHz)	Double labeling
	N	N	N
MGd	125	49	2 (1.1%)
MGm/SG	29	10	3 (6.7%)
MGv	154	73	12 (5.6%)
Total	308	132	17 (3.9%)

N is the number of labeled cells. The percent is the ratio of the number of double-labeled cells to total number of labeled cells.

*AAF* anterior auditory field, *MGd* dorsal subdivision of the MGB, *MGm/SG* medial subdivision of the MGB/suprageniculate nucleus, *MGv* ventral subdivision of the MGB.

## Discussion

**Figure 7:**
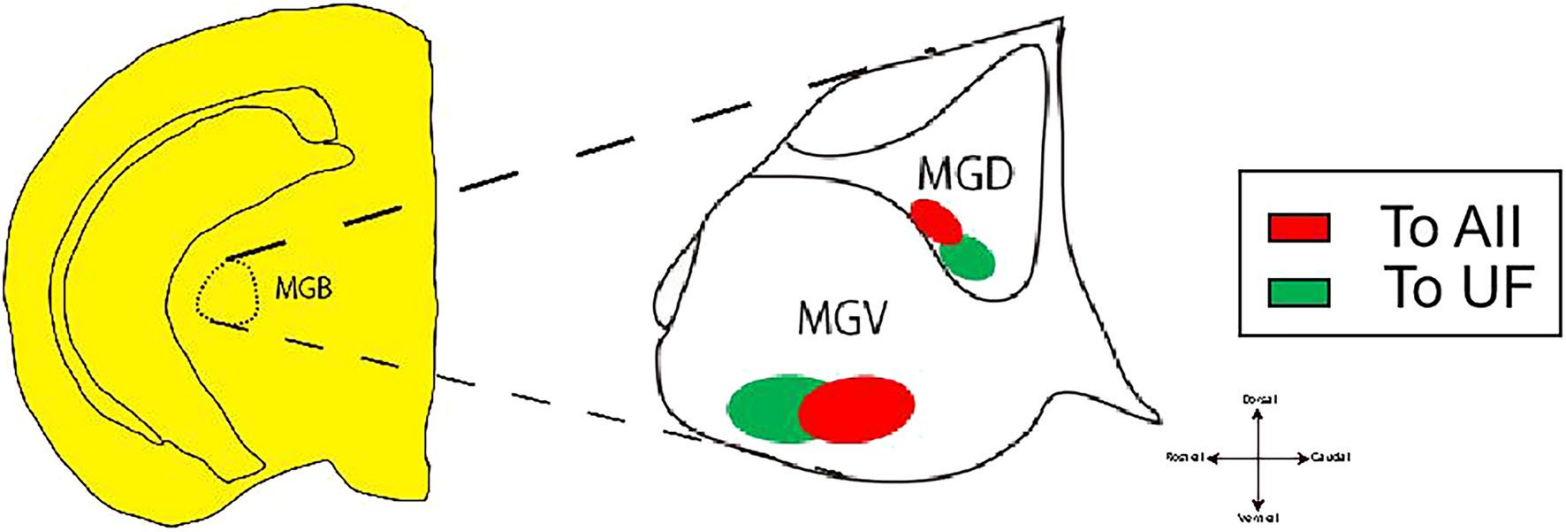
Schematic drawing of my research conclusion. (**A**) The figure shows that MGB subdivision project to UF and AII. Red part means neuron in ventral part of MGd and MGv project to AII; green part means neuron in ventral part of MGd and MGv project to UF.

In current study, we examined the projections from the identified belt areas (UF, AII and DP) in mice auditory cortex. These areas received input from MGB subdivision with independently manner. Not only that, some belt areas (UF, AII) received less common input from MGB with the largest core area (AAF). All these observations suggest that these pathways may convey auditory information in parallel (Fig. 7). Nevertheless, several double labeled cells were localized in the border between single-labeled cell clusters in the ventral part of MGv indicating that these neurons may project axons to both high-order core and belt area. Meanwhile these belt areas may cooperatively stream auditory information to sub-cortical structures.

Belt areas in mouse auditory cortex were less focused, which maybe caused by their small sizes. Combined high-resolution optical imaging with voltage sensitive dyebased retrograde labelling was essential for identifying the projection targets of belt areas. The thalamo-cortical projection of core area of mouse auditory cortex were examined in previous, but it is still unclear which parts of MGB project to belt areas. This may be due to the difficulty of accurate identification. Compared with mouse brain atlas identification, optical imaging maybe an accurate way for this experiment. This technique enabled to identify comprehensively three auditory core sub-fields (that is, AI, AAF, and IAF) based on their tonotopic gradients, and three belt sub-fields (that is, UF, AII, and DP) based on the relative positions of their OR sites to core areas (Fig. 1) in reference to the previous study^5,22,23^. Traditional tracer injection experiment usually use stereotaxic frame and brain atlas, but for small cortical belt area optical imaging may detect such area response more accurately and quickly for tracer injection experiment. Optical imaging not only accurate detected the position of belt areas, but also provide these small area is independently exist.

Otherwise, the AII-projection and DP projection does not seem to be completely the same compared with previous studies. Our results showed AII receiving projection from MGv and part of MGd; DP receiving projection from MGd and SG. I think these meticulous difference may be caused by the auditory sub-field detected method. Optical imaging detected tonotopy and relative position of AII were different with previous studies^10,28^. This difference may be caused by the difference of signal detecting mechanism. In vivo experiments, autofluorescence usually be used as an indirect measure of neuronal activity. Transcranial flavoprotein fluorescence imaging using the oxidation/reduction of flavoproteins to detect the fluorescence signal change between neuronal activity^29^. Optical imaging detecting the fluorescence that produced by dye stained brain surface directly. Some study indicates the fast signal that was detected by optical imaging may be more similar to electrophysiology^30^, so we choose optical imaging to identify the auditory sub-fields in mice. The meticulous difference of identified auditory sub-fields may evoked by these difference detected methods.

Some previous studies proposed that UF might be a part of core areas, since no gap was found in tonotopic organizations between UF and AAF^22,23,27^. In the results of our study, there were obvious boundaries between UF and AI or AAF, and also between AII or DP and AAF (Fig. 1A). Especially in 4-kHz frequency there was a small area between AAF and AI. We did not deny that UF is a higher-order area, but it is difficult to identify UF under high frequency stimulation because of the fluorescence effect of AAF and AII. So the position of UF was identified under 4-kHz frequency stimulation. Tracer injection experiments also demonstrated that there was almost no common thalamic input between AAF and UF cortical areas (Fig. 6). All these results indicates a belt areas may exist and receive MGB input in an independent manner. In additional, blood vessel pattern on cortical surface may affect the optical responses signals. To eliminate this possibility, 56 times optical imaging experiments were repeated with different animals. The results shows relative positions of OR sites were almost same in each trials.

Our results also suggest the ventral part of MGv projects to two belt areas (UF and AII but not DP). Some studies have reported that only core areas receive input from MGv in cats and rats, whereas the sources of belt areas are MGm and MGd^12,17,18^. And other studies finding that MGv neurons projecting to the IAF, AAF, and AI, respectively, belong predominantly to distinct populations suggests that the differential auditory tuning properties of neurons in the cortical fields might be attributable in part to differential thalamic input^22^. Our results also indicate that MGv cells projecting to the AII and UF are localized to the ventral part of the MGv. Such topography as well as the spatial segregation of the these thalamo-cortical pathways should have implications in the mechanisms for the development of the pathways. Our result agrees with a previous study demonstrating that MGv projects to UF^31^. The onset responses to pure tones in belt areas were later than those in core areas (Fig. 1). The result raises the possibility that belt areas are modulated by core areas probably. Taken together, not only core areas (IAF, AAF, and AI), but also belt areas (UF and AII) receive input from MGv.

Double retrograde labeling of MGv neurons projecting to the UF and AII resulted in double-labeled cells localized only in a small part of ventral MGv. The double-labeled cells in the ventral part of MGv can be attributed either to MGv cells that project to both the UF and AII, or to tracer spread to the other cortical sub-fields such as AAF or AI. Since the positions of UF and AII are very close to the AAF, the diffusion of a tracer may caused labeling of AAF cells in the MGv. Although this possibility cannot completely be excluded in this study, we must highlight that if any MGv neuron projecting to both the UF and AII, labeling by tracer injection into frequency-matched sites in the two fields, should be the most effective method to reveal such neurons. The double tracer injection experiments revealed that only a higher frequency part of AAF receives input from ventral part of MGv, commonly to UF and AII (Figs. 5, 6). The result may suggests that the ventral portion of MGv projects only to cortical regions responding to high frequency tone. And our results call for investigation into the high frequency corresponding in the MGv and functional specialization of MGv neurons, with the neuron’s target area in the cortex being identified. To test whether the ventral part of MGv responds only to higher frequency tone, electrophysiological recording of the neuronal responses to sound stimulation may be needed in the future study.

MGB is the colliculus which belongs to the posterior thalamus and is connected with the hypocolliculus through the hypocolliculus arm. The cell population in its deep surface is the relay nucleus of the lateral colliculus auditory fibers. The auditory radiation is composed of the fibers emitted by MGB, and the posterior capsule reaches the auditory cortex of the brain. Our data MGv–UF, MGv–AII, and MGd/SG–DP projections are virtually parallel suggest that belt areas in mice may responsible for different acoustic sensory function such as sound direction, sound strength, or sound frequency.

## Conclusion

Our study investigated thalamo-cortical neuron circuits in mice auditory belt areas by the methods of optical imaging and double tracer injections. We found that auditory belt areas (that is, UF, AII, and DP) receive MGB input primarily in an independent manner, and that UF and AII receive input mainly from ventral part of MGv and MGd (Fig. 7A) with a small subset of neurons localized at the ventral part of MGv projecting to both (UF and AII) areas. This founding may provides clues for the functional role of auditory cortical belt areas.

## Data Availability

All authors confirm that the data supporting the data and materials of this study are available within the article.
